# Increasing HPV and Cervical Cancer Education Among Native American Communities and Healthcare Providers

**DOI:** 10.1007/s13187-025-02619-w

**Published:** 2025-04-10

**Authors:** T. R. Joe, K. M. Natonie, K. R. Charley, N. R. Lee

**Affiliations:** 1 Interdisciplinary Health Program and Department of Health Sciences, Northern Arizona University, Flagstaff, AZ USA; 2 Department of Family & Community Medicine, University of Arizona, Tucson, AZ USA; 3 Department of Chemistry and Biochemistry, Northern Arizona University, Flagstaff, AZ USA

**Keywords:** Vignette, Intervention, Knowledge and attitudes, Vaccine

## Abstract

Human papillomavirus (HPV) is the most common sexually transmitted infection. Among Native Americans, HPV-associated cancers can lead health disparities in cervical cancer and increased rates of oral cancer in men. We designed a pilot study using a culturally tailored educational intervention aimed to improve knowledge and attitudes toward HPV and the vaccine among community members and healthcare providers in Northern Arizona. The one-hour intervention included in-person and online presentations, along with pre-and post-electronic surveys that assessed changes in knowledge and attitudes regarding HPV. The surveys featured demographic questions, true/false knowledge questions, and vignettes. Of the 67 participants in the intervention, the majority were females aged 18 to 55 years. About 32% identified as Native American, 47% as non-Hispanic White, and 8% as Hispanic. Students comprised the majority (46%) of participants, while healthcare providers, educators, staff, and social workers accounted for 25%. Attitudes towards HPV and the vaccine indicated that 93% of participants recognized the importance of catching up on vaccinations, though only 64% supported vaccination following a positive Pap test. Vignettes showed generally positive attitudes towards vaccination, with strong agreement on its importance and safety. Knowledge about HPV, the vaccine, and its association with cancer improved following the intervention. The pilot study demonstrates that culturally tailored educational interventions can effectively enhance knowledge and attitudes toward HPV and its vaccine, potentially reducing cancer-related disparities in Native American communities. Ongoing efforts are necessary to address remaining gaps in awareness and vaccine uptake.

**Supplementary Information:**

The online version contains supplementary material available at 10.1007/s13187-025-02619-w.

## Introduction

### Cervical Cancer in US and Native American Communities

Cervical cancer is one of the most common cancers affecting women, with an estimated 13,800 cases reported in 2020 [1]. American Indian/Alaska Native (herein referred to as Native American) women are disproportionately impacted by cervical cancer, exhibiting higher prevalence and mortality rates compared to non-Hispanic White women, with a prevalence rate 2.11 times higher and a mortality rate 1.55 times higher [2–5]. Cervical cancer can be attributed to persistent human papillomavirus (HPV) infections. Disparities in cervical cancer rates among Native American (NA) communities can be attributed to several factors, including a lack of screening, unequal access to healthcare, lower rates of HPV vaccination, a higher prevalence of oncogenic, or high-risk, HPV genotypes, and limited awareness and knowledge about HPV and its vaccine [6].

### HPV Vaccination and Prevalence in Native American Communities

Currently, Gardasil 9® is the only recommended HPV vaccine for both males and females in the U.S. The vaccine is recommended for individuals aged 11 to 16 years, but it can be administered as early as age 9, following a two-dose regimen. Adults aged 27 to 45 may also choose to receive the vaccine after consulting with their physician [7]. Since the introduction of the HPV vaccine, the prevalence of HPV types covered by the vaccine has declined among women in the USA. However, there is currently no approved test for detecting HPV in males. Additionally, it is unclear whether the prevalence of HPV among NA women has declined due to limited representation in national studies [8–11]. Previous research indicates that the decline may be attributed to the vaccine not covering certain prevalent HPV genotypes found among NAs, such as HPV types 51, 52, 56, and 68. These studies also show that NA women have higher rates of high-risk HPV infections compared to White women [10, 11].

### Factors Contributing to HPV Infections and Cervical Cancer in Native Americans

Primary prevention of HPV infections relies on vaccination and cancer screening. While vaccine initiation and series completion rates are higher among NAs compared to the general population, catch-up vaccination rates and cervical screening remain low. This contributes to a higher prevalence of HPV in this group [5, 12, 13]. Several factors contribute to these low rates, including challenges such as finding childcare, taking time off work, lack of transportation, negative experiences with medical care, mistrust of healthcare systems, feelings of fear or embarrassment related to pelvic exams, and cultural attitudes and taboos [5, 11, 14]. Moreover, a lack of knowledge about HPV, its associated cancers, and the vaccine intensifies these challenges [2, 5, 13–15]. One significant barrier is the limited information available to NA parents and concerns regarding the safety of the HPV vaccine. Research shows that vaccine uptake increases when parents are educated about the vaccine’s safety and its effectiveness in preventing HPV-associated cancers, particularly when healthcare providers recommend it. Our previous study revealed that even among professionals in STEM and health-related fields, there are gaps in knowledge about HPV and the vaccine [13].

In this pilot study, we adapted an existing educational intervention by developing a culturally tailored version [13]. We conducted an intervention study aimed at educating community members and healthcare providers who serve NA populations in Northern Arizona about HPV, the HPV vaccine, HPV-related diseases, recent findings on HPV, and its impact on NA women, as well as the CDC’s recommendations regarding HPV vaccination. Our overall and long-term goal is to reduce HPV-associated cancers in NA communities through vaccination, screening, and educational interventions focused on HPV and cancer.

## Methods

### Ethical Statement and Considerations

The study was Institutional Review Board (IRB) approved from Northern Arizona University (NAU IRB 1487466–1). Given the history of exploitative and unethical research, it was essential for researchers, including those of NA descent, to conduct the study ethically [16]. The educational intervention was culturally tailored, incorporating cultural relevance, sensitivity, and input from the community. The information provided about cancer and HPV was made pertinent to the local NA community, addressing associated health disparities while avoiding stigmatization. The focus of the intervention was on sensitive communication and empowering individuals to learn about cancer, HPV, and related recommendations through storytelling via vignettes. This method respected the traditional communication style used by NAs and aimed to avoid highlighting the negative experiences and statistics often associated with these topics. Additionally, the cultural practice of the presenter sharing a personal familial experience with cancer and identifying as a Native individual can help to establish trust, familiarity, and rapport with the participants from the beginning of the study.

The study recognized the historical mistrust surrounding medical research by being transparent from the start of the study. Participants were provided with information about study’s objectives, which included educating healthcare professionals and community workers about HPV and the vaccine. In addition, the study provided healthcare workers with the latest recommendations on HPV and their implications for NA women, as well as shared recent findings on HPV and its significance for this population. Furthermore, the study communicated how the findings could be applied in the future to enhance rates of screening and cancer prevention programs in Native communities. Importantly, there were no additional risks for participants associated with the addendum, and it was consistently emphasized that participation was voluntary.

### Study Design

The educational intervention study was modeled after a previous study, that analyzed knowledge of HPV and participant attitudes [13]. Therefore, the pilot study expanded the findings as part of the requirements for a Master of Public Health internship at the University of Arizona. The study included a 45–60-min in-person and online PowerPoint presentation, along with a pre-and post-electronic survey to assess participants’ knowledge and attitudes regarding HPV, the HPV vaccine, and HPV-related diseases (such as cancers and warts). Three vignettes were also presented, each with two prompts to measure participants’ responses using a 5-point Likert scale (Supp. Table 1). Initially, two in-person presentations were scheduled for January 2020. However, due to the global COVID-19 pandemic, the in-person sessions were canceled. An IRB addendum was requested and approved to allow presentations to be delivered via Zoom®.

### Study Participants

The inclusion criteria for study participants were individuals aged 18 years or older, from any racial or ethnic background, regardless of gender, and holding community roles such as college students, healthcare providers (including MD, PAs, and nurse), healthcare center staff, health educators, social workers, and community workers relevant to serving NA populations (referred to as “Other” in this study).

### Recruitment Strategy

Participant recruitment involved sending emails to four local organizations: The Partnership for Native American Cancer Prevention (NACP), Northern Arizona University (NAU) Applied Indigenous Studies class, Native Americans for Community Action (NACA), and North Country HealthCare (NCHC). These organizations serve NA populations. The emails explained that the purpose of the study was to educate healthcare providers and community workers about HPV and the vaccine, considering the existing disparities in cervical cancer rates within these communities [6]. Additionally, the emails encouraged healthcare providers and other healthcare staff members to participate in the study.

### Surveys

The electronic surveys were administered using Poll Everywhere®, an interactive platform that enables real-time feedback during PowerPoint presentations. Participation in the survey was optional, and responses were anonymous. No personal information, such as cellphone numbers or other digital identifiers, was collected. Participants were able to answer the survey questions using their personal devices through a provided Poll Everywhere® link or code. For those who could not use an electronic device, a paper version of the survey was available as an alternative to ensure maximum voluntary participation (*n* = 15). Both in-person and online presentations facilitated data collection through the survey embedded within the PowerPoint presentation. By participating, individuals consented to have their responses used in future publications and/or presentations.

### Vignettes

Three narrative-style vignettes, along with statements utilizing a 5-point Likert scale (strongly agree, agree, neither agree nor disagree, disagree, and strongly disagree) were used to assess participants’ attitudes toward HPV and the vaccine. These vignettes presented relatable and understandable stories, engaging participants effectively [17]. Throughout the presentation, the vignettes were accompanied by questions regarding knowledge. Vignettes provide a means to explore actions within context, clarify people’s judgments, and offer a less personal and less threatening approach to discussing the HPV vaccine. They also align with the traditional communication styles of many NA communities, using oral and visual storytelling methods that are both familiar and effective. Additionally, vignettes present a third-party perspective, which can be beneficial when discussing sensitive subjects like cancer or other difficult topics [17].

### Pre-/Post-Survey Questions

The survey consisted of seven TRUE/FALSE statements designed to evaluate participants’ knowledge of HPV, the vaccine, and HPV-related diseases or infections. Participants completed both pre-and post- survey questions to measure the knowledge they gained during the presentation. Educational content was provided after the pre-survey questions to help assess learning outcomes in the post-survey.

### Statistical Analysis

Data from organizations and partnerships were combined, and descriptive statistics, including frequencies and percentages, were calculated. Participant attitudes toward HPV and the HPV vaccine were visually analyzed using stacked bar charts. The percentages of TRUE and FALSE responses in the pre- and post-surveys were compared to determine which group had a higher proportion of correct responses. A greater proportion of correct responses indicated that the educational intervention had some measurable effect on knowledge gained. All data analyses were performed using MS Excel.

## Results

### Demographics

The study’s results were gathered from participants’ responses through four different presentations, with the overall demographics summarized in Table 1. The surveys had 67 respondents, 72% coming from NACP, 19% from NAU, 6% from NACA, and 3% from NCHC. In terms of demographics, participants identified as follows: 47% White non-Hispanic, 32% Native American/American Indian/Alaska Native, 8% Hispanic or Latino/a/x, and 4% each for Asian, Pacific Islander/Native Hawaiian, and African or African American. Regarding community roles, 46% of participants were students, 9% were healthcare providers and health educators, 5% were healthcare center staff, 2% were social workers, and 25% held other roles. The gender distribution was predominantly female, with 76% identifying as such, 22% as male, and 2% not specifying. Age-wise, the largest group consisted of participants aged 18–26 years (54%), followed by those aged 27–35 years (20%), 36–45 years (14%), 46–55 years (4%), over 55 years (7%), and 2% preferred not to answer.

**Table 1:** Demographic characteristics of educational intervention participants (*N* = 67^#^)

Characteristics	*N* responded	*n*	%
Organizations/partnerships	67		
The Partnership for Native American Cancer		48	71.6
Prevention			
Northern Arizona University		13	19.4
North Country Health Center		2	3.0
Native Americans for Community Action		4	6.0
Race/ethnicity*	72		
White, non-Hispanic		34	47.2
Native American, American Indian, Alaska		23	31.9
Native			
Hispanic, Latino/a/x or Chicano/a		6	8.3
Asian		3	4.2
Pacific Islander/Native Hawaiian		3	4.2
African or African American		3	4.2
Community Role	54		
Student (all levels)		26	48.1
Healthcare provider (physician, PA, nurse, etc.)		5	9.3
Health educator		5	9.3
Healthcare center staff		3	5.6
Social worker		1	1.9
Other		14	25.9
Gender	55		
Female		42	76.4
Male		12	21.8
Not listed		1	1.8
Age	56		
18–26 years		30	53.6
27–35 years		11	19.6
36–45 years		8	14.3
46–55 years		2	3.6
Over 55 years		4	7.1
Preferred not to answer		1	1.8

*Categories not mutually exclusive, add to > 100%; participants were allowed a max of 4 selections for race/ethnicity

^#^Not all respondents answered all questions. Total for each category does not equal to 67. All percentages are based on unique responses to each question

### Attitude Toward HPV and HPV Vaccine

**Fig. 1:**
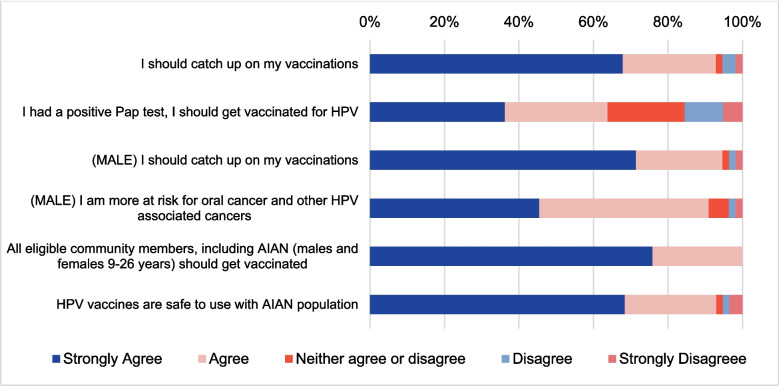
Participants’ attitudes towards HPV and HPV vaccination. Results above indicate that overall participants agree with positive aspects of the HPV vaccine education and the HPV connection to certain cancers. The first two prompts are related to Vignette #1: 24-year-old Native American (NA) college girl living in a college dorm and doesn’t have HPV vaccination; Participants agreed the woman should catch up on her HPV vaccination, and should get vaccinated after having a positive Pap test. The next two prompts are from Vignette #2: 24-year-old NA male with a family history of cancer and doesn’t have HPV vaccination. The last two prompts are from Vignette #3: NA woman with 4 children (ages 9–11) traveling a long distance to seek healthcare and has hesitancy against the HPV vaccine; despite the woman’s hesitancy participants generally agreed that HPV vaccines are safe should be accessible

Participants generally held positive views toward HPV and its vaccine, as illustrated in the vignette results (Fig. 1, Supp. Table 1). Vignette #1 featured a NA woman living in a college dorm, who had no history of receiving the HPV vaccine and showed no signs of HPV during Pap tests. About 68% of all participants strongly agreed, and 25% agreed that she should catch up on her vaccinations. However, when the woman had a positive Pap test for HPV infection, only 36% strongly agreed, and 28% agreed that she should receive the vaccine, whereas 21% neither agreed nor disagreed, indicating some hesitancy regarding the need for vaccination. Vignette #2 involved a NA male with a family history of cancer and no history of HPV vaccination, who visited a doctor for a sore throat and was recommended vaccination against HPV. Around 71% of participants strongly agreed, and 23% agreed that he should catch up on his vaccinations. Additionally, 45% strongly agreed and 45% agreed with the statement that he was at higher risk for oral cancer and other HPV-associated cancers. Vignette #3 featured a NA single mother with four children aged 9 to 11, who drove an hour to reach a clinic for checkups for two of her children. Although the doctor recommended that she begin vaccinating her children, she was reluctant to do so due to negative cultural stigma surrounding the HPV vaccine, financial struggles, and concerns about her children missing school, especially since the clinic is a long drive away. Despite her concerns, 76% of participants strongly agreed, and 24% agreed that all eligible community members, including NAs aged 9 to 26 years, should receive the vaccine. Furthermore, 93% of participants considered the HPV vaccines safe for use in NA populations, although a small minority, 5%, disagreed with its safety. These results underscore the consensus on the importance and safety of HPV vaccination while also highlighting areas where further education is needed to address lingering concerns.

### HPV and HPV Vaccine Knowledge Before and After Educational Intervention

Participants’ general knowledge of HPV and the vaccine varied based on the survey questions; however, following an educational intervention, their understanding improved. The overall average for correct answers on the pre-survey was 71.9%, while the post-survey showed a marked increase to 97.5%. A side-by-side percentage comparison of the survey responses before (pre-survey) and after (post-survey) the educational intervention is presented in Table 2. Each question regarding HPV knowledge yielded different correct response rates. Initially, 70.2% of participants correctly identified HPV as the most common sexually transmitted infection, but this increased to 100% after the intervention, demonstrating substantial improvement. Similarly, knowledge that there are more than 150 different types of HPV increased from 87.7 to 100%, indicating significant enhancement in understanding. The belief that all warts on the body are caused by HPV also saw a notable increase in correct responses, rising from 53.4 to 96.0%. Additionally, awareness that HPV causes more oral cancer in men improved from 73.3 to 100%. Misunderstandings about the HPV vaccine (Gardasil 9®) were effectively addressed, with correct responses regarding the number of high-risk HPV types the vaccine protects against increasing from 43.1 to 90.6%. The vaccine protects against seven high-risk types, while the other two types are low-risk types that cause warts. Furthermore, understanding that the HPV vaccine cannot cause HPV increased from 77.2 to 96.3%. Finally, awareness that NA women are at a higher risk for cervical cancer due to healthcare barriers and higher HPV prevalence was already high at 98.3% and increased to 100% post survey. Overall, these results indicate that the educational intervention effectively enhanced participants’ knowledge about HPV and its related issues.

**Table 2:** Comparison between electronic pre-survey and post-survey on knowledge of HPV, HPV vaccine, and HPV-related infections

Survey questions^b^	Pre-survey *n* (%)	Post-survey *n* (%)
HPV is the most common sexually transmitted infection		
Total number of participants (*N*)	57	52
TRUE^a^	40 (70.2)	52 (100.0)
FALSE	17 (29.8)	0 (0.0)
There are more than 150 different types of HPV		
Total number of participants (*N*)	57	53
TRUE^a^	50 (87.7)	53 (100.0)
FALSE	7 (12.3)	0 (0.0)
All warts on the body are caused by HPV		
Total number of participants (*N*)	58	50
TRUE^a^	31 (53.4)	48 (96.0)
FALSE	27 (46.6)	2 (4.0)
HPV causes more oral cancer in men		
Total number of participants (*N*)	60	51
TRUE^a^	44 (73.3)	51 (100.0)
FALSE	16 (26.7)	0 (0.0)
The HPV vaccine (Gardasil 9) protects against 9 HPV types that can cause cancer		
Total number of participants (*N*)	58	53
TRUE	33 (56.9)	5 (9.4)
FALSE^a^	25 (43.1)	48 (90.6)
The chance of contracting HPV from the HPV vaccine is ZERO		
Total number of participants (*N*)	57	54
TRUE^a^	44 (77.2)	52 (96.3)
FALSE	13 (22.8)	2 (3.7)
AIAN women are at a higher risk for cervical cancer due to the barriers in healthcare and higher prevalence of HPV		
Total number of participants (*N*)	58	53
TRUE^a^	57 (98.3)	53 (100.0)
FALSE	1 (1.7)	0 (0.0)

^a^Represents correct answers

^b^Questions are from previously published material by NRL: Lee et al. [13]

Two open-ended questions were posed regarding the types of treatments available for HPV infections and the recommended age for NA women to start getting Pap tests (individual answers not shown). Responses regarding treatment options included medications, vaccinations, the loop procedure, antibiotics, surgical procedures, or indicated that they did not know. This variety of responses suggests a misunderstanding of HPV, including its infection process, prevalence as a sexual transmitted infection, and how and when it clears from the body. As for the age at which NA women should begin receiving Pap tests, responses ranged from as young as 14 to as old as 30, with an average response of 21. Some participants noted that testing should start “when sexually active.” Overall, we can conclude that the participants possess a general understanding of when women should start getting Pap tests.

## Discussion

Studies have indicated a necessity for culturally tailored educational interventions focused on HPV, the HPV vaccine, and related HPV diseases within NA communities [13, 18, 19]. Currently, educational initiatives are limited; however, one intervention aimed at increasing vaccination coverage among NA girls has shown promising results [20]. In our culturally tailored intervention study, we evaluated the knowledge gained by students, Native-serving healthcare providers, and staff regarding HPV, the HPV vaccine, and HPV-related diseases. We also informed them of the current CDC recommendations for HPV vaccination.

The survey results regarding attitudes toward HPV and the HPV vaccine demonstrate generally positive views among all participants. There is a strong consensus on the importance of catching up on vaccinations and the safety of the HPV vaccine for the NA population. This understanding was reinforced by the general HPV education provided before participants answered the vignette questions. While the intervention has successfully improved attitudes toward vaccination, the varying levels of agreement across the vignettes suggest that more targeted strategies are needed. For instance, additional tailored educational intervention efforts might be necessary, especially concerning the benefits of HPV vaccination after a positive Pap test. It is also important to address the small fraction of participants who express concerns about the vaccine's safety. Furthermore, we need to address cultural misconceptions and fears to overcome hesitation in HPV vaccination uptake. These insights can help guide targeted interventions aimed at improving knowledge and addressing any misconceptions or concerns within the community, ultimately influencing post-intervention vaccination rates.

The educational intervention improved participants’ knowledge about HPV across survey questions, as evidenced by the higher percentage of correct answers in the post-survey. This improvement indicates that participants may be more inclined to seek HPV vaccination. Furthermore, with this enhanced understanding, individuals are better equipped to make informed and well-rounded decisions about HPV vaccination, including taking steps to prevent HPV-associated cancers and other related diseases or infections.

The pilot study is one of the first to investigate HPV knowledge and its impact on NA populations. It specifically focuses on educating individuals about HPV, the HPV vaccine, and HPV-related diseases, using pre- and post-survey questions and vignettes. Culturally tailored educational initiatives can play a crucial role in improving understanding and increasing vaccination rates among NA communities. The findings from this study can inform the development of more effective public health strategies that are responsive to the specific needs and cultural contexts of NA populations. Current ongoing efforts include educational interventions that utilize a text-message based system to follow up with participants and provide information about HPV, the HPV vaccine, and HPV-related diseases. Utilizing text messaging as a means of communicating this information, as well as for sending follow-up reminders, can be beneficial for participants who may travel long distances to receive healthcare.

Although the intervention successfully improved knowledge, barriers to increasing vaccination uptake persists, emphasizing the need for ongoing culturally tailored strategies. To effectively address knowledge gaps and misconceptions, future interventions can employ a multi-faceted approach that combines education with personalized support, follow-ups, and community-based initiatives. This strategy can help bridge the gap between acquiring knowledge and making actual behavior changes, ultimately leading to higher vaccination rates and reduced health disparities related to HPV-related cancers.

Future interventions should prioritize continuous education through follow-up sessions and reminders that emphasize the importance of HPV vaccination uptake. This can be achieved by implementing free vaccination-based incentive educational intervention programs or by engaging healthcare providers directly during the decision-making process to encourage and recommend vaccination. The long-term impact of these educational interventions can be enhanced by involving trusted community figures, such as community leaders, healthcare providers and staff, to build strong relationships and foster trust, ultimately reducing vaccine hesitancy. Additionally, outreach programs, flyers, and support from community leaders can contribute to lasting behavior changes regarding HPV vaccination uptake. Culturally tailored educational interventions should focus on assessing the effectiveness of these efforts on vaccination uptake and completion among NA teens and adults. Overall, the goal of educational interventions focused on HPV and related topics is to help reduce HPV-related health disparities and promote health equity within this population.

## Limitations

The pilot study involved 67 participants; thus, it is important to acknowledge its limitations. The relatively small sample size restricts the generalizability of the findings beyond this specific group of participants in Northern Arizona. Additionally, the two-semester timeline of the master’s internship, which included outreach, coordination, and the implementation of presentations, suggests that at least one more semester would have provided more opportunities for additional presentations and greater participation from individuals with various community roles related to health. A longer timeline may also have allowed for pursuing tribal IRB approval and inclusion of tribal health centers, which primarily serve NA populations and employ healthcare providers and staff who identify as NA. Furthermore, recruiting participants in collaboration with the Arizona Community Health Workers Association (AzCHOW) would be beneficial, as their community health workers provide health education directly to patients. The intervention was initially planned as an in-person event but had to be adapted to virtual presentations due to the ongoing COVID-19 pandemic. Since virtual technology for these presentations was new, participation may have decreased. However, this adaptation allowed individuals who may not have attended in person to participate online. These limitations underscore the need for cautious interpretation of the findings and highlight the importance of replicating the study with larger, population-specific participants.

## Conclusion

In conclusion, our pilot study highlights the urgent need for culturally tailored educational interventions that address HPV, the HPV vaccine, and associated diseases within NA populations. Previous research has shown that such interventions are often limited in availability, yet they improve vaccination uptake. Our findings suggest that these culturally tailored educational initiatives can play a vital role in enhancing understanding and increasing vaccination rates among Native-serving providers and the NA community.

## Supplementary Information

Below is the link to the electronic supplementary material.

ESM 1 (DOCX 19.9 KB)

## Data Availability

Raw data and the powerpoint presentation used in the intervention are available by the corresponding author.
