# Supplementary material for: Increasing HPV and Cervical Cancer Education Among Native American Communities and Healthcare Providers
Source: J Cancer Educ. 2025 Apr 10;41(1):40–8. doi: 10.1007/s13187-025-02619-w (PMC12971791; doi:10.1007/s13187-025-02619-w)
Supplement: Supplementary file 1 — (DOCX 19.9 KB) [file 13187_2025_2619_MOESM1_ESM.docx]

| **Supplementary Table 1. Raw 5-point Likert scale responses to vignettes and their associated prompts (N=67^#^)** | | | | | | | | |
| --- | --- | --- | --- | --- | --- | --- | --- | --- |
|  | **Prompts** | **N**  **Responded^*^** | **Strongly Agree** | **Agree** | **Neither agree or disagree** | **Disagree** | **Strongly Disagree** |  |
| Vignette #1 | I should catch up on my vaccinations | 56 | 67.9% | 25.0% | 1.8% | 3.6% | 1.8% |  |
|  | I had a positive Pap test, I should get vaccinated for HPV | 58 | 36.2% | 27.6% | 20.7% | 10.3% | 5.2% |  |
| Vignette #2 | (MALE) I should catch up on my vaccinations | 56 | 71.4% | 23.2% | 1.8% | 1.8% | 1.8% |  |
|  | (MALE) I am more at risk for oral cancer and other HPV associated cancers | 55 | 45.5% | 45.5% | 5.5% | 1.8% | 1.8% |  |
| Vignette #3 | All eligible community members, including AIAN (males and females 9-26 years) should get vaccinated | 58 | 75.9% | 24.1% | 0.0% | 0.0% | 0.0% |  |
|  | HPV vaccines are safe to use with AIAN population | 57 | 68.4% | 24.6% | 1.8% | 1.8% | 3.5% |  |
| ^#^Not all respondents answered the prompts, so the total for each category may not add up to 67. All percentages are based on unique responses to each prompt  ^*^Not every participant answered the prompts | | | | | | | | |
